# Changes and correlations of serum interleukins, adhesion molecules and soluble E-selectin in children with allergic rhinitis and asthma

**DOI:** 10.12669/pjms.345.15334

**Published:** 2018

**Authors:** Jing Bi, Yaoqin Hu, Zhaoyang Peng, He Liu, Yong Fu

**Affiliations:** 1 Jing Bi, Department of Otolaryngology Head and Neck Surgery, The Children’s Hospital, Zhejiang University School of Medicine, Hangzhou 310052, Zhejiang Province, China; 2 Yaoqin Hu, Department of Anesthesiology, The Children’s Hospital, Zhejiang University School of Medicine, Hangzhou 310052, Zhejiang Province, China; 3 Zhaoyang Peng, Medical Testing Center, The Children’s Hospital, Zhejiang University School of Medicine, Hangzhou 310052, Zhejiang Province, China; 4 He Liu, Department of Otorhinolaryngology, Beijing Aerospace General Hospital, Beijing 100076, China. The Children’s Hospital, Zhejiang University School of Medicine, Hangzhou 310052, Zhejiang Province, China; 5 Yong Fu, Department of Otolaryngology Head and Neck Surgery, The Children’s Hospital, Zhejiang University School of Medicine, Hangzhou 310052, Zhejiang Province, China

**Keywords:** Adhesion molecule, Allergic rhinitis, Asthma, E-selectin, Interleukin

## Abstract

**Objective:**

To observe the changes and correlations of serum interleukins (ILs), adhesion molecules and soluble E-selectin (sE-selectin) in children with allergic rhinitis, asthma and both diseases.

**Methods:**

A total of 45 children with allergic rhinitis, 40 with asthma and 45 with allergic rhinitis complicated with asthma treated from September 2016 to January 2018 were selected. Meanwhile, 30 healthy subjects who received physical examinations were included as a control group. The levels of serum IL-4, IL-5, IL-10, soluble intercellular adhesion molecule-1 (sICAM-1), soluble vascular adhesion molecule-1 (sVCAM-1), and sE-selectin were detected by double-antibody sandwich ELISA, and their correlations were subjected to Spearman’s correlation analysis.

**Results:**

The serum IL levels of allergic rhinitis, asthma and complication groups were significantly higher than those of control group (P<0.01), and the levels of complication group significantly exceeded those of asthma group (P<0.05). The serum levels of IL-5 and IL-10 in complication group significantly exceeded those of allergic rhinitis group (P<0.05). Compared with control group, serum sICAM-1, sVCAM-1, and sE-selectin levels significantly increased in other three groups (P<0.01). Such levels of complication group were significantly higher than those of allergic rhinitis and asthma groups (P<0.05). Serum IL-10 level was positively correlated with that of IL-4 (r=0.965, P<0.05), and sE-selectin level was positively correlated with those of sICAM-1 and sVCAM-1 (r=0.915, P<0.01; r=0.892, P<0.01).

**Conclusion:**

Serum IL-4, IL-5, IL-10, adhesion molecules and sE-selectin are all involved in the pathogenesis of allergic rhinitis and asthma, which can be used to evaluate the degrees of respiratory allergic diseases.

## INTRODUCTION

Allergic asthma is one of the common diseases that seriously threaten the health of pediatric respiratory system. About 300 million people in the world are suffering from allergic asthma, and the incidence is about 9.6% and 8.9% in the United States and Sweden respectively.^1^ A clinical epidemiological study in China showed that the incidence of allergic asthma in children was approximately 7.57%-48%.^2^ With social development and industrialization, the incidence and death rates of pediatric allergic asthma have increased annually. The incidence of asthma in children under 14 years old range between 0.12% and 3.34%^3^, with allergy being one of the main causes.^4^ Notably, 30%-90% of children with allergic asthma are complicated with allergic rhinitis^5^ which has become one of the related factors for onset of the former^6^, thereby directly increasing the frequency of attack, hospitalization rate and economic burden.

According to clinical observations, allergic rhinitis often precedes asthma, and the treatment for asthmatic patients with severe allergic rhinitis is tricky. Pathological and physiological studies also suggest that rhinitis and asthma are closely related. Although rhinitis and asthma are different, the upper and lower respiratory tracts may be affected by a common inflammatory process at the same time, and this inflammatory response can persist or develop through an interconnected mechanism. An allergic disease may be systemic. Allergen challenge tests of both the upper and lower respiratory tracts can cause inflammatory responses at the other end of the respiratory tract. Adhesion molecules participate in and mediate the adhesion of multiple inflammatory cells and transendothelial metastasis. The roles of intercellular adhesion molecules (ICAMs) in the pathogenesis of asthma have attracted wide attention.^7^,^8^ Similarly, adhesion molecules have also been found to play crucial roles in the pathogenesis of allergic rhinitis. Li YZ et al. reported that the expressions of inflammatory CAMs in vascular endothelial cells, glandular cells, submucosal lymphocytes, and eosinophils in the nasal mucosal tissues of patients with allergic rhinitis were significantly higher than those of the healthy control group.^9^ Adhesion molecules can be detached from the cell surface and exist in serum in soluble forms. They may be proteolytically lysed from the cell surface and released into the extracellular fluid to form serum soluble ICAM-1 (sICAM-1), soluble vascular intercellular adhesion molecule-1 (sVCAM-1), soluble E-selectin (sE-selectin), etc.

It has previously been reported that the levels of soluble adhesion molecules in serum can reflect those of CAMs in the airway and lungs.^10^ Thus, the expressions of CAMs therein can be indirectly determined by detecting serum soluble adhesion molecules in patients with allergic rhinitis and asthma. Interleukin-4 (IL-4), IL-5, and IL-10 are the main cytokines secreted by Th2 lymphocytes and eosinophils. Their levels may indirectly indicate the degrees of inflammatory cell activation, differentiation and proliferation. The different levels in patients with rhinitis, asthma, and allergic rhinitis complicated with asthma may suggest various degrees of inflammatory cell infiltration. In this study, the levels of soluble adhesion molecules and ILs in serum were detected by double-antibody sandwich ELISA. The inflammatory responses of children with allergic rhinitis, asthma, and asthma complicated with allergic rhinitis were revealed by measuring the above-mentioned indices, aiming to provide a theoretical basis for studying the pathogenesis of asthma and proposing preventive and therapeutic strategies.

## METHODS

A total of 130 children diagnosed and treated in our hospital from September 2016 to January 2018 were selected. There were 40 asthmatic children, including 18 boys and 22 girls aged between three and 12 years old, (8.4±1.8) on average. The cases were diagnosed in accordance with the Bronchial Asthma Prevention Guidelines formulated by the Asthma Group of the Respiratory Society, Chinese Medical Association.^11^ There were 45 children with rhinitis, including 26 boys and 19 girls aged between three and 12 years old, (8.1±1.6) on average. The cases were diagnosed according to the Guidelines for Diagnosis and Treatment of Pediatric Allergic Rhinitis stipulated by the Otolaryngology Head and Neck Surgery Branch of Chinese Medical Association.^12^ Another 45 children who suffered from allergic rhinitis complicated with asthma were included, comprising 21 boys and 24 girls aged between three and 12 years old, (8.1±1.8) on average. These cases were diagnosed in accordance with both guidelines. Neither hormones nor antihistamines were taken in the past five weeks. Meanwhile, 30 healthy children who received physical examinations in our hospital during the same period were selected as a control group, including 14 boys and 16 girls aged between three and 12 years old, (8.4±1.4) on average. All children had no history of diseases other than allergic rhinitis and asthma. This study has been approved by the hospital’s ethics committee, and written consent has been obtained from the guardians of selected children.

### Detection methods

Before blood collection, all subjects were fasted for 12 hour. Then blood samples were collected through the cubital veins in the early morning and centrifuged to take serum for detection. IL-4, IL-5, and IL-10 were detected using serum IL kits (Shanghai Research Chemicals Co., Ltd., China). sICAM-1 and sVCAM-1 were detected with adhesion molecule kits (Shanghai Times Biotechnology Co., Ltd., China). sE-selectin was detected using a sE-selectin kit (Shanghai Times Biotech Co., Ltd., China). All detections were performed in strict accordance with the kits’ instructions.

### Statistical analysis

All data were analyzed by SPSS 16.0 and expressed as Ax ± s. Given the heterogeneity of variance, inter-group pair wise comparisons were performed by the rank-sum test with completely randomly designed multiple sample comparison. Correlations between indices were subjected to Spearman’s correlation analysis. P<0.05 was considered statistically significant.

## RESULTS

### Serum levels of IL-4, IL-5 and IL-10

The serum levels of IL-4 in control, allergic rhinitis, asthma and complication groups were (5.23±2.78), (82.17±32.45), (69.42±36.19) and (80.65±33.01) ng/L, respectively. The IL-5 levels of allergic rhinitis, asthma and complication groups were 12.2, 9.9 and 14.2 times that of the control group (3.14 ±2.75) ng/L), respectively. The serum levels of IL-10 in allergic rhinitis, asthma and complication groups were 9.9, 10.0 and 12.7 times that of the control group (6.15±2.98) ng/L), respectively (Table-I). The serum IL levels of allergic rhinitis, asthma and complication groups were significantly higher than those of the control group (P<0.01), and the levels of the complication group significantly exceeded those of the asthma group (P<0.05). The serum levels of IL-5 and IL-10 in the complication group were significantly higher than those of the allergic rhinitis group (P<0.05).

**Table-I:** Serum levels of IL-4, IL-5 and IL-10 (ng/L, x̄±s)

Group	Case No.	IL-4	IL-5	IL-10
Control	30	5.23±2.78	3.14±2.75	6.15±2.98
Allergic rhinitis	45	82.17±32.45^a^	38.29±19.74^a^	60.79±34.68^a^
Asthma	40	69.42±36.19^a^	30.96±16.85^a^	61.27±28.95^a^
Complication	45	80.65±33.01^ac^	44.63±12.62^abc^	78.06±24.31^abc^

Compared with control group,

a P<0.01; compared with allergic rhinitis group,

b P<0.05; compared with asthma group,

c P<0.05. IL: Interleukin.

### Levels of serum adhesion molecules and sE-selectin

The serum levels of sICAM-1 in control, allergic rhinitis, asthma and complication groups were (338.61±74.95), (598.82±257.36), (520.54±189.43) and (627.09±226.87) μg/L, respectively. The sVCAM-1 levels of allergic rhinitis, asthma and complication groups were all about 1 time that of the control group, while the sE-selectin levels of the three groups were 2.2, 1.8 and 3.2 times that of the control group, respectively (Table-II). Compared with the control group, serum sICAM-1, sVCAM-1, and sE-selectin levels significantly increased in the other three groups (P<0.01). Such levels of the complication group were significantly higher than those of allergic rhinitis and asthma groups (P<0.05).

**Table-II:** Levels of serum adhesion molecules and sE-selectin (μg/L, x±s)

Group	Case No.	sICAM-1	sVCAM-1	sE-selectin
Control	30	338.61±74.95	342.75±72.86	5.87±1.69
Allergic rhinitis	45	598.82±257.36^a^	669.74±196.15^a^	12.91±6.32^a^
Asthma	40	520.54±189.43^a^	657.58±168.76^a^	10.59±5.85^a^
Complication	45	627.09±226.87^abc^	799.84±202.18^abc^	18.97±6.29^abc^

Compared with control group,

a P<0.01; compared with allergic rhinitis group,

b P<0.05; compared with asthma group,

c P<0.05. sICAM-1: soluble intercellular adhesion molecule-1; sVCAM-1: soluble vascular adhesion molecule-1; sE-selectin: soluble E-selectin.

### Results of rank-sum test with completely randomly designed multiple sample comparison for serum IL-4, IL-5 and IL-10

The IL-4, IL-5 and IL-10 levels of allergic rhinitis, asthma and complication groups were significantly different from those of the control group (P<0.01) (Table-III).The IL-5 levels of allergic rhinitis and asthma groups were significantly different (P<0.01). Allergic rhinitis and complication groups had significantly different IL-5 and IL-10 levels (P<0.05, P<0.01), and the asthma group had significantly different ILs levels from those of the complication group (P<0.01, P<0.01, P<0.05).

**Table-III:** Results of rank-sum test with completely randomly designed multiple sample comparison for serum IL-4, IL-5 and IL-10.

Control group	IL-4	q	IL-5	q	IL-10	q
Allergic rhinitis and control groups	2501.0	10.6**	2304.0	13.0**	2165.0	12.4**
Asthma and control groups	1975.0	16.8**	1802.5	15.5**	2010.5	17.5**
Complication and control groups	2447.5	14.2**	2720.0	11.6**	2682.5	12.0**
Allergic rhinitis and asthma groups	499.5	2.6	504.5	4.2**	156.5	1.5
Allergic rhinitis and complication groups	32.5	0.3	418.0	3.8*	530.5	4.7**
Asthma and complication groups	471.0	4.1**	1002.0	5.3**	684.0	4.0*

* P<0.05,

** P<0.01. IL: Interleukin.

### Results of rank-sum test with completely randomly designed multiple sample comparison for serum adhesion molecules and sE-selectin

The sICAM-1, sVCAM-1 and sE-selectin levels were significantly different between groups, except for those between allergic rhinitis and asthma groups or allergic rhinitis and complication groups (P<0.01) (Table-IV). The three levels of allergic rhinitis and complication groups were significantly different (P<0.05), whereas allergic rhinitis and asthma groups only had significantly different sE-selectin levels (P<0.01).

**Table-IV:** Results of rank-sum test with completely randomly designed multiple sample comparison for serum adhesion molecules and sE-selectin.

Control group	sICAM-1	q	sVCAM-1	q	sE-selectin	q
Allergic rhinitis and control groups	1210.5	6.8**	1902.0	15.9**	1802.0	10.0**
Asthma and control groups	890.0	7.6**	2047.0	11.7**	1130.5	9.6**
Complication and control groups	1635.0	7.0**	2525.0	10.8**	2175.5	9.2**
Allergic rhinitis and asthma groups	322.0	2.9	152.0	1.3	674.0	5.7**
Allergic rhinitis and complication groups	435.5	3.7*	627.5	3.7*	376.0	3.3*
Asthma and complication groups	754.0	4.4**	481.0	4.2**	1051.0	6.1**

* P<0.05,

** P<0.01. sICAM-1: soluble intercellular adhesion molecule-1; sVCAM-1: soluble vascular adhesion molecule-1; sE-selectin: soluble E-selectin.

### Correlation between serum ILs, adhesion molecules and sE-selectin

The results of Spearman’s correlation analysis showed that serum IL-10 level was positively correlated with that of IL-4 (r=0.965, P<0.05), and sE-selectin level was positively correlated with those of sICAM-1 and sVCAM-1 (r=0.915, P<0.01; r=0.892, P<0.01).

## DISCUSSION

The reason for the coexistence between allergic rhinitis and asthma is the interconnected nostrils with the respiratory bronchus in terms of body structure. When the allergen stimulates one of them, it is easy to cause lesions in related parts. Allergic rhinitis and asthma have very similar infiltration of inflammatory cells, such as eosinophils. The presence of inflammatory cells, such as eosinophils and neutrophils, around the airways is promoted by substances like adhesion molecules. The inflammatory cells then secrete some products so as to cause inflammatory reactions. We studied the changes in inflammatory cells in patients with allergic rhinitis, asthma, and allergic rhinitis complicated with asthma, such as serum ILs and adhesion molecules, to find out the similarities, differences and relations between these diseases, so as to provide a factual basis for revealing the pathogenesis and effective treatment of allergic rhinitis and asthma.

We found that compared with the control group, three serum ILs, i.e. IL-4, IL-5 and IL-10, two adhesion molecules, i.e. sICAM-1 and sVCAM-1, and sE-selectin were significantly higher in the allergic rhinitis group, the asthma group and the allergic rhinitis combined with asthma group (P<0.01), indicating that these indices play an important role in the pathogenesis of allergic rhinitis and asthma. Consistently, there are differences in the serum adhesion molecules and ILs between children with allergic rhinitis and asthma,^13^ and IL- 4 and IL-5 change in children with allergic rhinitis and asthma.^14^ In addition, the serum levels of IL-4, IL-5, IL-10, sICAM-1, sVCAM-1, and sE-selectin in the combined group were significantly higher than those in the asthma group (P<0.05), indicating that the inflammatory status of the former group was lower than that of the latter group. IL-4, IL-5, and IL-10 are secretions of Th2 cells, whose overexpression can lead to an imbalance of proportion between Th1 and Th2 cells and promote the conversion of Th2 cells so as to aggravate the conditions.^15^-^17^ Moreover, the levels of IL-5 and IL-10, adhesion molecules, and sE-selectin in the combined group were significantly higher than those in the allergic rhinitis group and the asthma group (P<0.05). According to the literature, this may be associated with allergic factors and genetic factors.^18^-^20^ Through completely randomly designed rank sum test for pair-wise comparative analysis, the results showed that serum levels of IL-4, IL-5, IL-10, sICAM-1, sVCAM-1, and sE-selectin 6 in the allergic rhinitis group, the asthmatic group, and the combined group were higher than those of the control group, and the results of the combined group were higher than those of the asthma group. Except for IL-4, the other five factors in the combined group were higher than those in the allergic rhinitis group, further proving that allergic rhinitis combined with asthma inflammation is more severe than allergic rhinitis and asthma. The results of Spearman’s analysis showed that serum IL-10 was positively correlated with IL-4, and sE-selectin was positively correlated with sICAM-1 and sVCAM-1, suggesting that IL-10 and sE-selectin accelerate the development of allergic rhinitis and asthma.

In summary, serum IL-4, IL-5 and IL-10, adhesion molecules, and sE-selectin were all involved in the pathogenesis of allergic rhinitis and asthma, which can be used to diagnose the degree of respiratory allergic diseases, thus being potentially applicable to clinical practice.

### Authors’ Contributions

**JB and YF** designed this study and prepared this manuscript.

**YH, ZP and HL** performed this study and analyzed clinical data.
